# Supplementary material for: Degradation of red blood cell deformability during cold storage in blood bags
Source: EJHaem. 2021 Nov 24;3(1):63–71. doi: 10.1002/jha2.343 (PMC9176030; doi:10.1002/jha2.343)
Supplement: Supplementary file 1 — Figure S1. Cumulative distribution curves after deformability‐based sorting of RBCs using the microfluidic ratchet device. (A) Cumulative distribution curves from deformability sorting of RBC units at week 0 (day of manufacturing, blue line), followed by weeks 2 (green), 4 (orange), 6 (red), and 8 (black) of cold storage. Each donor showed distinct RBC deformability loss profiles (shift to the right) during storage. [file JHA2-3-63-s001.pdf]

Supplemental Information for

Degradation of Red Blood Cell Deformability during Cold Storage in Blood Bags

Authors: Emel Islamzada, Kerryn Matthews, Erik Lamoureux, Simon P. Duffy, Mark D. Scott, Hongshen Ma

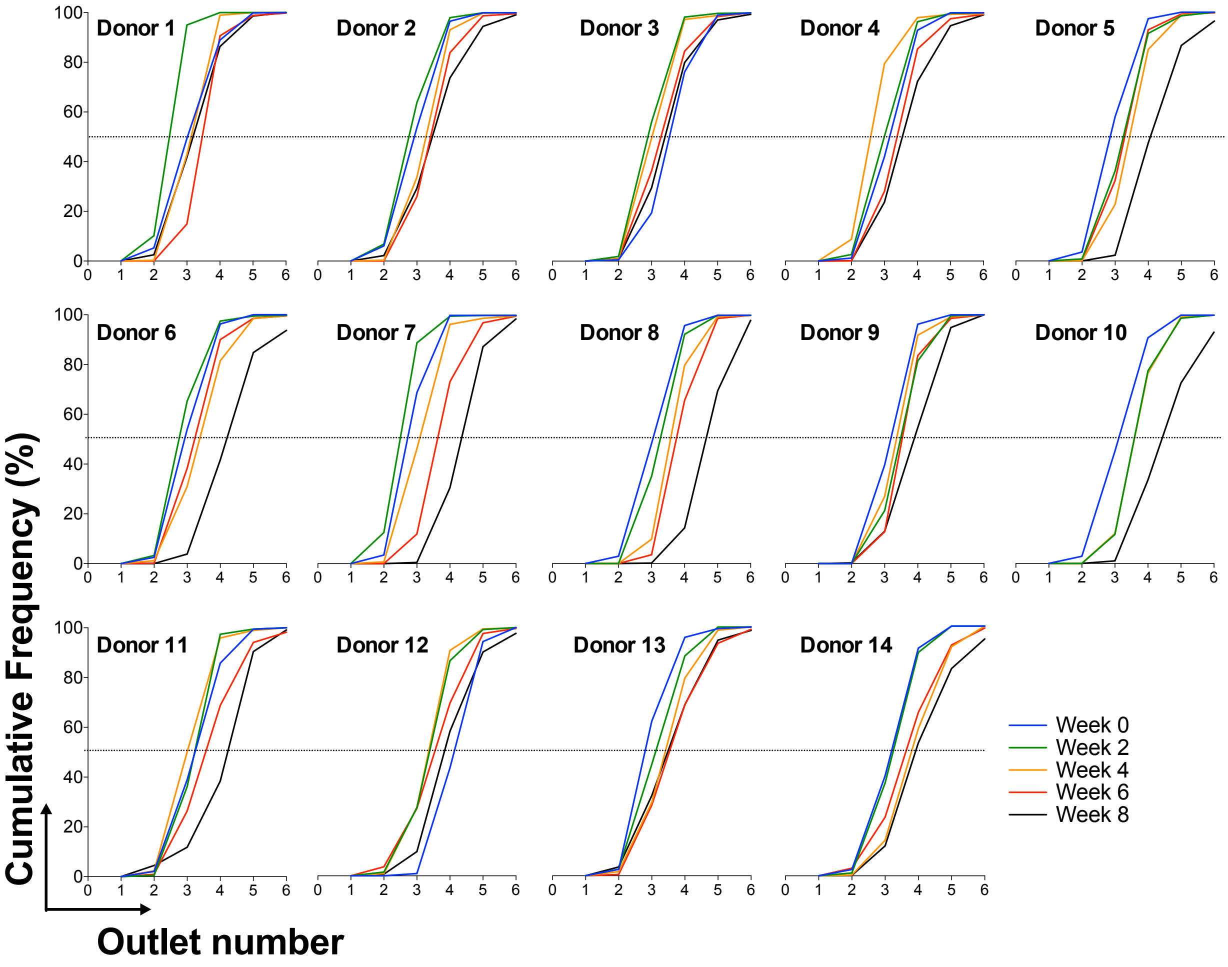

Figure S1. Cumulative distribution curves after deformability-based sorting of RBCs using the microfluidic ratchet device. (A) Cumulative distribution curves from deformability sorting of RBC units at Week 0 (day of manufacturing, blue line), followed by Weeks 2 (green), 4 (orange), 6 (red), and 8 (black) of cold storage. Each donor showed distinct RBC deformability loss profiles (shift to the right) during storage.
